# The impact of chronic kidney disease on outcomes following peripheral vascular intervention

**DOI:** 10.1002/clc.23444

**Published:** 2020-08-11

**Authors:** Dennis I. Narcisse, Elizabeth Hope Weissler, Jennifer A. Rymer, Ehrin J. Armstrong, Eric A. Secemsky, William A. Gray, Jihad A. Mustapha, George L. Adams, Gary M. Ansel, Manesh R. Patel, William Schuyler Jones

**Affiliations:** ^1^ Division of Cardiology, Department of Medicine Duke University Health System Durham North Carolina USA; ^2^ Division of Cardiology Duke Clinical Research Institute Durham North Carolina USA; ^3^ Division of Vascular and Endovascular Surgery, Department of Surgery Duke University Health System Durham North Carolina USA; ^4^ Cardiovascular Systems Inc St. Paul Minnesota USA; ^5^ Division of Cardiology Rocky Mountain Regional VA Medical Center, University of Colorado Denver Colorado USA; ^6^ Division of Cardiology Beth Israel Deaconess Medical Center Boston Massachusetts USA; ^7^ Division of Cardiology Main Line Health, Lankenau Heart Institute Wynnewood Pennsylvania USA; ^8^ Division of Cardiology Advanced Cardiac and Vascular Centers forAmputation Prevention Grand Rapids Michigan USA; ^9^ Division of Cardiology UNC REX Healthcare Raleigh North Carolina USA; ^10^ Division of Cardiology OhioHealth Heart and Vascular Physicians Columbus Ohio USA

**Keywords:** chronic kidney disease, mortality, peripheral artery disease, revasculariation

## Abstract

**Background:**

Patients with chronic kidney disease (CKD) have worsened clinical outcomes following percutaneous coronary intervention; however, limited evidence exists in patients undergoing peripheral vascular intervention (PVI).

**Purpose:**

We aimed to assess the effect of CKD on outcomes following PVI for symptomatic peripheral artery disease.

**Methods:**

Using patients from the LIBERTY 360 study, we compared the rates of 30 day and 1 year major adverse vascular events (MAVE), a composite of all‐cause mortality, major amputation, and target vessel/lesion revascularization, between patients with and without CKD (estimated glomular filtration rate less than 60) following PVI. Multivariable adjustment was performed to assess for independent association between CKD and outcomes.

**Results:**

Among 1189 patients enrolled, 378 patients (31.8%) had CKD. At 1 year, patients with CKD had higher rates of MAVE (34.6% vs 25.6%), all‐cause mortality (11.9% vs 5.5%), and major amputation (5.9% vs 2.6%) when compared with patients without CKD (all *P* < .05). After adjustment, patients with CKD had higher risks of 1‐year MAVE (HR 1.30, 95% CI 1.04‐1.64; *P* = .023) and all‐cause mortality (HR 1.88, 95% CI 1.22‐2.91; *P* = .005) when compared with patients without CKD. There was no statistically significant difference in risk of major amputations (HR 1.70, 95% CI 0.91‐3.17; *P* = .094).

**Conclusions:**

Despite high procedural success and low amputation rates, patients with CKD remain at greater risk for MAVE and all‐cause mortality after PVI. Further research is needed to determine treatment strategies to mitigate substantial mortality risk in this vulnerable population.

## 1 INTRODUCTION

Peripheral artery disease (PAD) is a manifestation of systemic atherosclerosis leading to malperfusion of the lower extremities, which can cause debilitating symptoms and/or tissue loss. Despite an increasing prevalence, PAD remains underappreciated by both patients and clinicians as a significant risk factor for cardiovascular morbidity and mortality.^1^ Peripheral revascularization is frequently performed to improve symptoms among claudicants and reduce the risks of major lower extremity amputation among those with chronic limb threatening ischemia. Endovascular revascularization has been utilized as the preferred strategy in most patients, yet little is known about risk factors and clinical outcomes following peripheral vascular intervention (PVI).^2^

Chronic kidney disease (CKD) is a known risk factor for PAD, and patients with CKD often present with significant calcification, more severe disease, and longer lesions.^3^, ^4^ Despite this higher burden of disease, there have been reports that patients with CKD are offered fewer overall revascularization procedures and more frequently suffer major amputations.^5^ Indeed, some studies have suggested that after PVI, CKD is independently associated with poor outcomes, including higher rates of death and amputation.^6^ There has also been a stronger association seen with more severe renal disease.^6^, ^7^, ^8^, ^9^ However, evidence remains limited on the best approach for these complex medical patients.

The LIBERTY 360 study prospectively enrolled patients with symptomatic PAD undergoing PVI, captured discrete data elements at the time of PVI, and collected clinical outcomes. In order to add to the limited knowledge about success and outcomes of PVI in patients with CKD, we aimed to describe patient‐ and procedure‐related characteristics of patients with and without CKD and characterize the impact of CKD on major cardiovascular events and ischemic limb outcomes following PVI.

## 2 METHODS

### 2.1 Study design and oversight

LIBERTY 360 is a prospective, observational, multicenter study that was performed to examine predictors of clinical outcomes in patients with distal lower extremity PAD who underwent PVI of the distal superficial femoral, popliteal, and/or tibial arteries. The study design, rationale, and data analysis for LIBERTY 360 have been previously published.^10^, ^11^ Clinical follow‐up was performed at 30 days, 6 months, 12 months, and 2 years; patients were followed up to 5 years. A steering committee consisting of LIBERTY 360 principal investigators, representatives from the study core laboratory, and the sponsor (Cardiovascular Systems, Inc) were responsible for development of the original study protocol, which was approved by the IRB at each study site. The study was registered on ClinicalTrials.gov (NCT01855412).

### 2.2 Study population

Patients were enrolled if they were above the age of 18 years, presented with symptomatic PAD (at least Rutherford class 2), and had an indication for peripheral revascularization. Patients were included if they had revascularization of a stenosis located in the distal superficial femoral artery, popliteal artery, or tibial arteries (specifically any lesion distal to a point 10 cm above the medial epicondyle of the femur). Lesions needed to be present within a native vessel, be traversable with a guidewire, and be treated with an FDA‐approved endovascular device. Exclusion criteria included: conversion from endovascular intervention to surgical revascularization, in‐stent restenosis in all lesions in the target area, and an expected life span of less than 1 year. CKD was determined by the case report forms completed by investigators; estimated glomular filtration rate (eGFR) was measured separately by sites' labs and recorded in the database. CKD staging was not recorded in the case report form. Patients were excluded if their CKD status was unable to be determined.

### 2.3 End points

There were multiple prespecified outcomes in the observational LIBERTY 360 study. The primary outcome of our analysis was the rate of major adverse vascular events (MAVE), defined as a composite endpoint including all‐cause mortality, unplanned major amputation of the target limb, and clinically driven target vessel and/or lesion revascularization (TVR/TLR) at 30 days and 1 year. Major amputation was further defined as any unplanned major amputation of the target limb after the index procedure. TVR/TLR was defined as any revascularization, endovascular or surgical, of target vessel and/or lesion after index procedure. Reinterventions on the target limb at locations other than the index vessel or lesion were not captured. Acute limb ischemia was also not captured.

The secondary outcomes of this analysis consisted of rates of each individual MAVE component at 30 days and 1 year, in addition to procedural success after the index procedure and change in quality of life from baseline. Procedural success was defined as less than 50% residual stenosis for treated lesions without significant angiographic complications (flow‐limiting dissections [type C‐F], perforation, slow/no reflow, distal embolization, or abrupt closure). Adjudication of angiographic data was performed by SynvaCor/Prairie Educational and Research Cooperative (PERC; Springfield, IL). Quality of life was measured using the EQ‐5D Visual Analog Score (VAS) at baseline and each follow‐up visit.^12^

### 2.4 Statistical analysis

Categorical variables were compared between patients with CKD vs those without CKD using a Monte Carlo Approximation of the Fisher's Exact Test. Continuous variables were compared with ANOVA tests and for discrete continuous variables, *P*‐values were calculated from a Kruskal‐Wallis test. For the primary and secondary outcomes, Kaplan–Meier time‐to‐event methodology was used to estimate unadjusted event rates through each time point; Greenwood's method used to obtain the 95% confidence interval for the estimate. A Cox proportional hazards model was analyzed controlling for the baseline characteristics of age, gender, race, body mass index, smoking status, comorbid conditions (coronary artery disease [CAD], hypertension, hyperlipidemia, diabetes, prior myocardial infarction [MI], prior stroke), Rutherford classification, ankle brachial index (ABI), and PAD history (prior endovascular treatment, bypass, or amputation on target limb). An adjusted multivariable model was created based on a Cox proportional hazards model using stepwise selection with an entry criterion from the univariable model of 0.15 and a stay criterion of 0.05 (Supplemental Table 1). The hazard function was then used to estimate the survival function. Confidence intervals are based on back‐transformed log‐log of the survival function. P‐values were calculated using Cox proportional hazards model for estimates at a specified time point. Imputation of significant angiographic complications for procedural success of core lab identified lesions were performed by using site data when the core lab was unable to perform angiographic assessment. Statistical analysis was performed by NAMSA (Northwood, OH) with input from the sponsor of the study. All data analysis was performed with the SAS Software System (SAS institute, Inc., Cary, NC).

## 3 RESULTS

**FIGURE 1:**
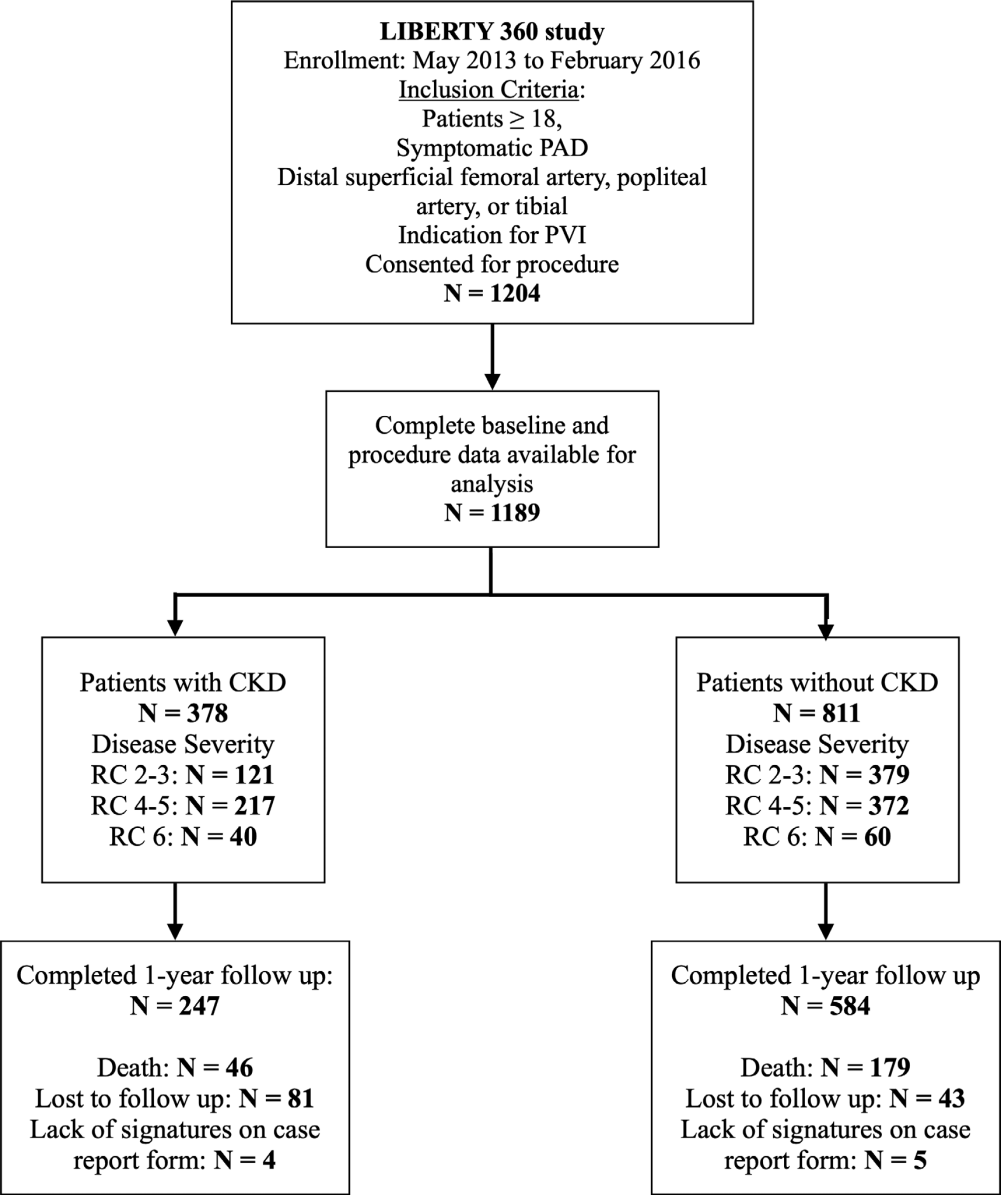
Ascertainment of the study population. CKD, patients with chronic kidney disease; PAD, peripheral artery disease; PVI, peripheral vascular intervention; RC, Rutherford classification

From May 2013 to February 2016, 1204 patients who underwent PVI for symptomatic PAD were enrolled; 1189 had complete baseline and procedure data available for analysis. A total of 378 patients (31.8%) had CKD. The median eGFR was 38.1 mL/min/1.73 m^2^ (IQR 21.9, 49.1) for patients with CKD and 72.9 mL/min/1.73 m^2^ (IQR 60.9, 85.5) for those without CKD. There were 87 (23.0%) of patients with CKD who were hemodialysis dependent. There were 15 patients excluded from the secondary analysis due to unknown CKD status. Follow‐up was available in 247 patients with CKD (65.3%) and 584 patients without CKD (72.0%) at 1 year (Figure 1).

### 3.1 Baseline and PAD‐specific characteristics

Table 1 shows the baseline patient characteristics of patients with and without CKD. When compared with patients without CKD, those with CKD were more often African American (18.3% vs 13.4%; *P* = .0360) and more frequently had diabetes mellitus (72.0% vs 56.1%; *P* < .0001), CAD (69.0% vs 57.5%; *P* = .001), a prior history of MI (28.8% vs 21.7%; *P* = .0086), and had never smoked (38.1% vs 29.1%; *P* = .0021). Patients with CKD more frequently had Rutherford 5 disease (33.9% vs 21.7%; *P* < .001) and non‐compressible ABIs (18.7% vs 8.3%; *P* < .001) at baseline. There were no differences in prior limb revascularization attempts, prior amputation rates of the target limb (6.9% vs 4.2%; *P* = .0634) or of the contralateral limb (8.2% vs 5.8%; *P* = .1313) between the groups. In both patients with and without CKD, prior ipsilateral amputations were almost exclusively at the level of the toes (93.8% and 95.2% of prior ipsilateral amputations, respectively, *P* = 1.00). Patients with CKD who had undergone amputation of the contralateral limb were less likely to have had below‐ or above‐knee amputations compared to patients without CKD (30.1% vs 65.5% of prior contralateral amputations, *P* = .002). However, patients with CKD had higher rates of prior amputations on both limbs (5.8% vs 1.0%; *P* < .0001). The median EQ‐5D VAS at baseline was significantly lower among patients with CKD (60.0, IQR 50.0, 79.0) than among patients without CKD (70.0, IQR 50.0, 80.0; *P* < .0001).

**TABLE 1:** Baseline characteristics

	With CKD (n = 378)	Without CKD (n = 811)	*P* value
*Demographics*			
Age (years), median^a^	71 (63, 80)	70 (63, 77)	.0988
Male	64.6	64.9	.9480
Race			
White	78.3	83.4	.0436
African American	18.3	13.4	.0360
Ethnicity			
Hispanic or Latino	15.6	13.6	.3726
BMI (kg/m^2^), median (IQR)	28.9 (25.6, 32.9)	27.9 (24.4, 32.1)	.0068
eGFR (mL/min/1.73 m^2^), median (IQR)^a^	38.1 (21.9, 49.1)	72.9 (60.9, 85.5)	<.0001
Hemodialysis dependent	23.0	0.0	.0003
*Cardiovascular risk factors*			
Diabetes mellitus	72.0	56.1	<.0001
Hypertension	96.3	92.0	.0056
Hyperlipidemia	88.6	86.1	.2321
Coronary artery disease	69.0	57.5	.0001
Previous history of MI	28.8	21.7	.0086
Previous history of CVA	16.7	14.2	.2948
Tobacco use			
Current smoker	10.1	23.2	<.0001
Former smoker	51.9	47.7	.1912
Never	38.1	29.1	.0021
*PAD history*			
Prior PVI			
Target limb	32.5	29.2	.2499
Contralateral limb	35.4	34.6	.7941
Prior LE surgical bypass			
Target limb	2.9	4.4	.2632
Contralateral limb	3.4	3.9	.7460
Prior amputation			
Target limb	6.9	4.2	.0634
Contralateral limb	8.2	5.8	.1313
Both limbs	5.8	1.0	<.0001
Highest level of amputation, target limb^b^			
Toe(s) only	93.8	95.2	1.000
Foot only	6.3	4.8	1.000
Highest level of amputation, contralateral limb^c^			
Toe(s) only	69.8	47.3	.0203
Foot only	11.3	7.3	.5230
Below knee/above ankle	22.6	47.3	.0090
Above the knee	7.5	18.2	.1513
*Rutherford classification*			
2	4.8	9.7	.0030
3	27.2	37.0	.0010
4	23.5	24.2	.8272
5	33.9	21.7	<.001
6	10.6	7.4	.0727
*ABI target limb* ^d^			
Abnormal ABI (<=0.90)	53.7	62.9	.0041
Borderline ABI (>0.90 to <1.00)	8.9	9.5	.8225
Normal ABI (>=1.00 to <=1.40)	18.7	19.2	.8682
Noncompressible	18.7	8.3	<.001
*Postprocedure medications*			
Aspirin	78.6	81.0	.3481
Clopidogrel	69.0	77.9	.0012
Prasugrel	4.8	2.6	.0555
Dual antiplatlet therapy	65.3	68.9	.2305
Anticoagulants	13.5	8.8	.0138
Lipid lowering therapy	82.5	78.1	.0766

*Note*: Values are % unless otherwise specified.

Abbreviations: ABI, ankle brachial index; BMI, body mass index; CKD, chronic kidney disease; CVA, cerebrovascular accident; eGFR, estimated glomerular filtration rate; IQR, interquartile range; LE, lower extremity; MI, myocardial infarction; PAD, peripheral artery disease; PVI, peripheral vascular intervention.

^a^ Age and eGFR missing for one patient without CKD (N = 810).

^b^ Percentage denominators based on number of target limb amputation data available: With CKD N = 48 and without CKD N = 42.

^c^ Percentage denominators based on number of contralateral limb amputation data available: With CKD N = 53 and without CKD N = 55.

^d^ Percentage denominators based on number of ABI data available: With CKD N = 337 and without CKD N = 769.

### 3.2 Lesion and procedural characteristics

Table 2 highlights the anatomy and lesion characteristics of patients at the time of the index procedure. There were 489 lesions treated in patients with CKD (1.3 ± 0.6 per patient) and 1039 lesions in those without CKD (1.3 ± 0.6 per patient). When compared with patients without CKD, patients with CKD more frequently had isolated infrapopliteal disease (57.5% vs 48.5%; *P* = .0012). Patients with CKD more frequently had zero patent runoff vessels (12.7% vs 8.2%; *P* = .0151) and more lesions that were severely calcified when compared to those without CKD (65.3% vs 55.4%; *P* = .0005). There were no differences between the groups when assessing mean target lesion length, degree of stenosis, and number of chronic total occlusions. The use of balloon angioplasty and stenting were similar between the patients with and without CKD. Atherectomy was commonly used in the study cohort, however it was used less frequently in patients with CKD (64.1% vs 71.3%; *P* = .0060) compared to those without CKD. Procedural success and complication rates did not significantly differ between the two groups (78.6% in patients with CKD vs 77.7% in patients without, *P* = .7541).

**TABLE 2:** Lesion and procedural characteristics

	With CKD (N = 378)	Without CKD (N = 811)	*P* value
*Lesion characteristics*			
Lesions treated	N = 489	N = 1039	
Lesions treated per patient^a^	1.3 ± 0.6	1.3 ± 0.6	.6874
Target lesion location			
ATK only	30.5	37.6	.0070
ATK and BTK	11.9	13.8	.3307
BTK only	57.5	48.5	.0012
Runoff vessels, pre‐procedure	N = 378	N = 809	
3	14.3	18.7	.0697
2	36.2	35.1	.7448
1	36.8	38.1	.6998
0	12.7	8.2	.0151
Degree of stenosis, pre‐procedure %^b^	82.2 ± 18.9	81.9 ± 19.9	.8318
Predominately calcified lesion^c^	65.3	55.4	.0005
Chronic total occlusion^d^	38.8	39.3	.8643
Target lesion length, mm^e^	116.6 ± 107.6	109.5 ± 105.6	.2379
< 40 mm	31.0	30.9	.9509
40‐99 mm	24.8	30.2	.0434
≥ 100 mm	44.1	39.0	.0722
*Procedural characteristics*			
Number of lesions	489	1039	
Lesion treated with balloon^f^	97.5	96.7	.4260
POBA	77.8	83.1	.0157
DCB	7.1	10.3	.0454
Lesion treated with atherectomy^f^	64.1	71.3	.0060
Lesion treated with stent^f^	14.9	18.0	.1623
DES	5.6	5.5	.9042
BMS	10.4	12.3	.3025
Procedural success^g^	78.6	77.7	.7541
Degree of stenosis, post‐procedure %^h^	32.9 ± 19.2	31.9 ± 19.5	.3723
Change in runoff patency post‐PVI, target limb^i^			
Worsened	1.6	2.8	.4553
No change	69.6	71.9	.5016
Improved	28.9	25.3	.3001
Significant angiographic complications^j^	8.0	8.8	.7341
Severe dissection (type C‐F)^k^	2.7	2.1	.5408
Perforation^k^	1.0	1.3	.5653
Slow/no reflow^k^	1.9	1.1	.2866
Abrupt closure^k^	1.0	1.3	.5775
Distal embolization^l^	3.9	5.4	.4526

*Note*: Values are % or mean ± SD. Procedural success is defined as less than 50% residual stenosis of lesions treated in a patient with no major significant angiographic complications.

Abbreviations: ATK, above the knee; BMS, bare metal stent; BTK, below the knee; CKD, chronic kidney disease; DCB, drug‐coated balloon; DES, drug‐eluting stent; POBA, plain old balloon angioplasty; PVI, peripheral vascular intervention.

^a^ Mean based on patients with lesion counts available: With CKD N = 376 and without CKD N = 808.

^b^ Mean based on lesions with stenosis data available: With CKD N = 474 and without CKD N = 1014.

^c^ Percentage denominator based on lesion data available: With CKD N = 447 and without CKD N = 971.

^d^ Percentage denominator based on lesion data available: With CKD N = 474 and without CKD N = 1014.

^e^ Percentage denominator based on lesion data available: With CKD N = 451 and without CKD N = 975.

^f^ Percentage denominator based on device information available from site: With CKD N = 482 and without CKD N = 1023.

^g^ Percentage denominator based on number of patients with procedure data available: With CKD N = 341 and without CKD N = 766.

^h^ Mean based on lesions with stenosis data available: With CKD N = 462 and without CKD N = 981.

^i^ Percentage denominator based on number of patients with post procedure runoff data available: With CKD = 253 and without CKD 538.

^j^ Percentage denominator based on number of patients with available data: With CKD N = 357 and without CKD N = 787.

^k^ Percentage denominator based on number of patients with available data: With CKD N = 361 and without CKD N = 791.

^l^ Percentage denominator based on number of patients with available data: With CKD N = 357 and without CKD N = 786.

### 3.3 Event rates in patients with CKD and without CKD

**FIGURE 2:**
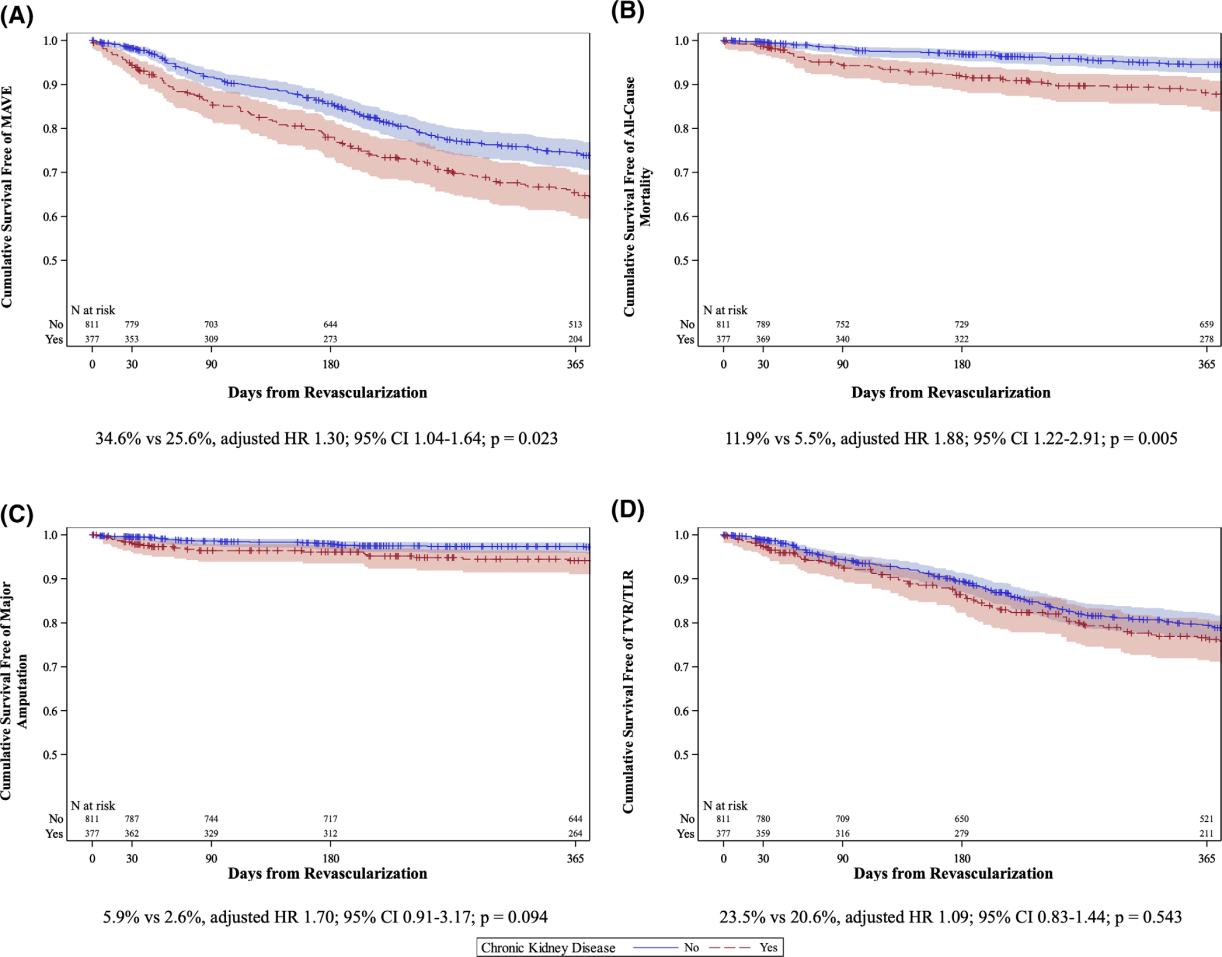
Kaplan–Meier curves comparing major Adverse vascular events, all‐cause mortality, major amputation, and target vessel/lesion revascularization in patients with and without chronic kidney disease. Kaplan Meier curves are shown for A, MAVE; B, all‐cause mortality; C, major amputation; and D, target vessel/lesion revascularization in patients with and without CKD. Event rates of each outcome for patients with CKD compared with patients without CKD at 1 year after PVI. CKD, chronica kidney disease; MAVE, major adverse vascular events; PVI, peripheral vascular intervention

Table 3 shows the event rates in patients with and without CKD. When compared with patients without CKD, patients with CKD had higher rates at 30 days of the primary composite end point MAVE (5.6% vs 1.7%; HR 3.27, 95% CI 1.66‐6.43; *P* = .006) and major amputation (1.9% vs 0.5%; HR 3.78, 95% CI 1.11‐12.92; *P* = .0339, Figure 1). At 1 year, patients with CKD had higher rates of MAVE (34.6% vs 25.6%; HR 1.47, 95% CI 1.17‐1.84; *P* = .0009), all‐cause mortality (11.9% vs 5.5%; HR 2.29, 95% CI 1.49‐3.52; *P* = .0002), and major amputation (5.9% vs 2.6%; HR 2.24, 95% CI 1.21‐4.17; *P* = .0107) compared with patients without CKD. Figure 2 depicts the Kaplan event rate curves for MAVE and its individual components.

**TABLE 3:** Association between CKD and cardiovascular and limb outcomes

	Unadjusted				Adjusted	
	With CKD	Without CKD	Univariable		Multivariable	
	(N = 377)	(N = 811)	HR (95% CI)	*P* Value	HR (95% CI)	*P* Value
30‐day MAVE	5.4%	1.7%	3.27 (1.66, 6.43)	.0006	2.52 (1.27, 4.98)	.008
All‐cause mortality	1.3%	0.4%	3.58 (0.86, 14.98)	.0807	3.17 (0.74, 13.46)	.119
Major amputation	1.9%	0.5%	3.78 (1.11, 12.92)	.0339	3.41 (0.99, 11.71)	.052
TVR/TLR	2.7%	1.1%	2.41 (0.98, 5.92)	.0561	2.01 (0.82, 4.98)	.129
1‐year MAVE	34.6%	25.6%	1.47 (1.17, 1.84)	.0009	1.30 (1.04, 1.64)	.023
All‐cause mortality	11.9%	5.5%	2.29 (1.49, 3.52)	.0002	1.88 (1.22, 2.91)	.005
Major amputation	5.9%	2.6%	2.24 (1.21, 4.17)	.0107	1.70 (0.91, 3.17)	.094
TVR/TLR	23.5%	20.6%	1.18 (0.90, 1.56)	.2287	1.09 (0.83, 1.44)	.543

Abbreviations: CI, confidence interval; CKD, chronic kidney disease; HR, hazard ratio; MAVE, major adverse vascular events (composite endpoint of all‐cause mortality, major amputation, and target vessel and/or limb revascularization [TVR/TLR]).

After adjustment for baseline variables, the presence of CKD was associated with 30‐day MAVE (HR 2.52, 95% CI 1.27‐4.98; *P* = .008), but not all‐cause mortality (HR 3.17, 95% CI 0.74‐13.46; *P* = .119) or major amputation (HR 3.41, 95% CI 0.99‐11.71; *P* = .052) compared with patients without CKD. However, at 1 year, patients with CKD had higher adjusted risks of MAVE (HR 1.30, 95% CI 1.04‐1.64; *P* = .023) and all‐cause mortality (HR 1.88, 95% CI 1.222.91; *P* = .005), but not major amputation (HR 1.70, 95% CI 0.91‐3.17; *P* = .094).

There were no differences in the median change in EQ‐5D VAS from baseline between patients with CKD and without CKD at 30 days (median change 3.0 in CKD and 5.0 in non‐CKD, *P* = .8481) or 12 months (median change 5.0 in both groups, 0.8121).

## 4 DISCUSSION

In this study, we examined the association of CKD with MAVE, consisting of all‐cause mortality, major amputation, and TVR/TLR, after endovascular intervention in the multicenter LIBERTY 360 study. Nearly one‐third of the 1204 symptomatic patients with PAD had a history of CKD. The unadjusted rates of MAVE among patients with CKD were higher at 30 days and 1 year following the initial intervention. This was largely driven by the increased rate of all‐cause mortality. After adjustment, patients with CKD still had greater risk of MAVE at both time intervals, as well as higher all‐cause mortality at 1 year. While patients with CKD experienced more major amputations at 30 days and 1 year, the adjusted risk was not statistically significant compared with those without CKD. Our findings underscore the substantial residual risk patients with CKD face post‐PVI despite good procedural outcomes. Indeed, these findings also highlight that more work needs to be done to improve on these outcomes.

Though the relationship between CKD and PAD is well established, our analysis contributes to the more limited data on whether PVI modifies the association between PAD, CKD, and all‐cause mortality. There have been a few retrospective analyses that have suggested that patients with CKD undergoing PVI had increased risks of cardiovascular mortality and ischemic limb events.^7^, ^8^, ^9^ However, in our analysis, the increased rate of the MAVE was driven by elevated rate of all‐cause mortality with low overall rates of major amputation. Thus, only all‐cause mortality at 1 year remained significant after adjustment. In other words, PVI may lower the likelihood of amputation in both groups, but patients with CKD retain a substantial residual risk of death not modified by revascularization. Prior studies have also found that CKD was a strong independent risk factor for death, with heightened risk associated with worse renal function.^5^ Similarly, in a study of veteran patients undergoing PVI, those with CKD had higher rates of mortality and more frequent progression to dialysis compared to patients without CKD undergoing PVI.^13^ While our study does suggest that the mortality risks of patients with PAD and concomitant CKD may not be mediated by PVI, the kidney‐specific risks of the procedure remain unclear in LIBERTY due to the lack of renal‐specific follow‐up. Using outcomes across many hospitals with high procedural success in both cohorts, it is clear that CKD contributes an inherent risk of higher cardiovascular mortality as a nature of the underlying disease and is an independent predictor of death post‐PVI.

Patients with CKD in our study did have higher unadjusted rates of major amputation when compared with patients without CKD and had several factors that are known to elevate the risk of major amputation after PVI. Advanced age, coexisting diabetes mellitus, advanced disease with higher Rutherford classification and fewer runoff vessels, and nonncompressible ABIs were all more frequent among patients with CKD in our analysis and all have been associated with worse limb‐related outcomes.^14^, ^15^, ^16^, ^17^ Also, the patients with CKD were more likely to have lesions treated below the knee than were patients without CKD, which has previously been shown to increase the risk of limb loss.^17^ Given that many of the factors associated with higher amputation rate were included in our model, the lack of significant association between CKD and major amputation after adjustment was unexpected. However, with a low overall rate of major ampuations, as evidenced by broad confidence interval estimated at 30 day (0.99‐11.71) and 1 year (0.91‐3.17), the lack of statistical significance may reflect a lack of power. It is also possible that the low amputation rates among both groups reflects contemporary practice and advanced technique in PVI. Ultimately, it remains prudent to continue working toward minimizing the amputation risk faced by patients with CKD following PVI.

Given our findings of poor outcomes in patients with CKD following PVI, the critical question is how to improve outcomes in this complex population. The literature has provided few alternative management strategies for patients with symptomatic PAD and concomitant CKD. These patients may benefit more from aggressive medical management with high‐intensity statins and/or PCSK9 inhibitors, more aggressive control of diabetes mellitus with GLP‐1 and/or SGLT2 inhibitors, and more aggressive antiplatelet or antithrombotic medications (including rivaroxaban as demonstrated in the COMPASS trial).^18^ These therapies have proven benefit and are often underprescribed in the PAD population.^19^, ^20^ Of the patients with CKD in our study, 78.6% were on aspirin, 82.5% on lipid lowering therapy, 69.0% on clopidogrel, and 65.3% on dual antiplatelet therapy post index procedure. Adherence to guideline recommended therapy should remain the major focus in the care of this patient population. Given the worse post‐PVI outcomes associated with CKD, there has been a trend towards lower rates of revascularization, both surgical and endovascular, even in patients with only moderate renal insuffiency.^5^ Our findings add context to the current limited knowledge of cardiovascular and limb outcomes following PVI, and it is clear that further work is needed in the CKD population. Specifically, a deficit of evidence exists about whether intervention is beneficial in patients with CKD and how to balance the kidney‐specific risks of PVI. It remains imperative to find the best approach to reduce symptom burden while minimizing the risk of mortality and limb loss in this vulnerable group.

### 4.1 Study limitations

Our study does have some limitations. The LIBERTY 360 study was observational in nature: the choices of PVI procedure type and devices were clinician‐ and site‐dependent, which may contribute to the more frequent rate of atherectomy use in LIBERTY than has been reported in Medicare analyses of office‐based laboratories.^21^ While this may decrease the generalizability of our findings, the frequency of atherectomy use in office‐based practice is increasing rapidly. Because this is a post‐hoc analysis, the original study was not powered for our analysis and did not collect all variables of relevance to studying CKD, including change in eGFR or need for dialysis over the course of follow‐up. Similarly, while target lesion and vessel revascularization were available, target limb revascularization was not collected, which may have lead to undercounting reinterventions. Finally, a relatively high number of patients were lost to follow up or withdrew over 1 year.

## 5 CONCLUSIONS

In conclusion, our results reflect contemporary practice of patients with symptomatic PAD with good procedural outcomes. After adjustment for baseline variables, CKD was independently associated with greater risk of all‐cause mortality at 1 year post‐PVI. There were low overall amputation rates and no significant difference in risk of amputation between the groups at 1 year. The increased risk of mortality among CKD patients suggests that PVI is not effectively mitigating the risk of death, and aggressive medical therapies with proven mortality benefit should likely be the focus in this vulnerable population. Ultimately, there is a need for more investigation into benefit and timing of endovascular intervention in patients with any degree of CKD.

## CONFLICT OF INTEREST

D. I. N., E. H. W.: They declare no potetntial conflict of interest. J. A. R.: Salary support from the American College of Cardiology. Research support from Boston Scientific and Abbott. E. J. A.: Consultant/Advisory Board: Abbott Vascular, Boston Scientific, Cardiovascular Systems, Gore, Intact Vascular, Medtronic, Philips. E. A. S.: Research grants to BIDMC: AstraZeneca, BD Bard, Boston Scientific, Cook Medical, CSI, Medtronic, Philips, and UCSF. Consulting: BD Bard, CSI, Medtronic, and Philips. Speaking Bureau: BD Bard, Cook Medical and Medtronic. W. A. G.: Research support: Boston Scientific, Medtronic, Surmodics, Gore, Intact Vascular, Philips. J. A. M.: Consultant to BD, Boston Scientific, Cardiovascular Systems, Inc., Medtronic, Philips, and Terumo. G. L. A.: Research support: Cardiovascular Systems, Inc., Bostom Scientific, Medtronic, Abbott Vascular, Philips, Gore, Cook Medical, BD Bard. Consulting: Cardiovascular Systems, Inc., Bostom Scientific, Medtronic, Abbott Vascular, Philips, Gore, Cook Medical, BD Bard. G. M. A.: Consulting: Cardiovascular Systems, Inc., Medtronic, Boston Scientific, Phillips, Surmodics. Royalties: Cook Medical. M. R. P.: Research grants from AHRQ, AstraZeneca, Bayer, Jansen, Procyrion, Heartflow; Honoraria/advisory board for Bayer, Janssen, AstraZeneca. W. S. J.: Research grants from Agency for Healthcare Research and Quality, AstraZeneca, American Heart Association, Bristol‐Myers Squibb, Doris Duke Charitable Foundation, Patient‐Centered Outcomes Research Institute; Honorarium/other from American College of Radiology, Daiichi Sankyo.

## FUNDING

The LIBERTY study and this sub‐analysis were funded by Cardiovascular Systems, Inc.

## Supporting information


**Supplemental Table 1** Analysis of covariates used in adjusted model for MAVE

## References

[1] Song P , Rudan D , Zhu Y , et al. Global, regional, and national prevalence and risk factors for peripheral artery disease in 2015: an updated systematic review and analysis. Lancet Glob Health. 2019;7(8):e1020‐e1030. 31303293 10.1016/S2214-109X(19)30255-4

[2] Simons JP , Schanzer A , Flahive JM , et al. Survival prediction in patients with chronic limb‐threatening ischemia who undergo infrainguinal revascularization. Eur J Vasc Endovasc Surg. 2019;58(1S):S120‐S134. 31151867 10.1016/j.ejvs.2019.04.009

[3] Liew YP , Bartholomew JR , Demirjian S , Michaels J , Schreiber MJ Jr . Combined effect of chronic kidney disease and peripheral arterial disease on all‐cause mortality in a high‐risk population. Clin J Am Soc Nephrol. 2008;3(4):1084‐1089. 18337552 10.2215/CJN.04411007 PMC2440260

[4] Foley RN , Murray AM , Li S , et al. Chronic kidney disease and the risk for cardiovascular disease, renal replacement, and death in the United States Medicare population, 1998 to 1999. J Am Soc Nephrol. 2005;16(2):489‐495. 15590763 10.1681/ASN.2004030203

[5] O'Hare AM , Bertenthal D , Sidawy AN , Shlipak MG , Sen S , Chren MM . Renal insufficiency and use of revascularization among a national cohort of men with advanced lower extremity peripheral arterial disease. Clin J Am Soc Nephrol. 2006;1(2):297‐304. 17699220 10.2215/CJN.01070905

[6] Lacroix P , Aboyans V , Desormais I , et al. Chronic kidney disease and the short‐term risk of mortality and amputation in patients hospitalized for peripheral artery disease. J Vasc Surg. 2013;58(4):966‐971. 23769941 10.1016/j.jvs.2013.04.007

[7] Patel VI , Mukhopadhyay S , Guest JM , et al. Impact of severe chronic kidney disease on outcomes of infrainguinal peripheral arterial intervention. J Vasc Surg. 2014;59(2):368‐375. 24176634 10.1016/j.jvs.2013.09.006

[8] Heideman PP , Rajebi MR , McKusick MA , et al. Impact of chronic kidney disease on clinical outcomes of endovascular treatment for Femoropopliteal arterial disease. J Vasc Interv Radiol. 2016;27(8):1204‐1214. 27321888 10.1016/j.jvir.2016.04.036 PMC4958546

[9] Xie JX , Glorioso TJ , Dattilo PB , et al. Effect of chronic kidney disease on mortality in patients who underwent lower extremity peripheral vascular intervention. Am J Cardiol. 2017;119(4):669‐674. 28027725 10.1016/j.amjcard.2016.10.053

[10] Adams GL , Mustapha J , Gray W , et al. The LIBERTY study: design of a prospective, observational, multicenter trial to evaluate the acute and long‐term clinical and economic outcomes of real‐world endovascular device interventions in treating peripheral artery disease. Am Heart J. 2016;174:14‐21. 26995365 10.1016/j.ahj.2015.12.013

[11] Mustapha J , Gray W , Martinsen BJ , et al. One‐year results of the LIBERTY 360 study: evaluation of acute and midterm clinical outcomes of peripheral endovascular device interventions. J Endovasc Ther. 2019;26(2):143‐154. 30722718 10.1177/1526602819827295 PMC6431778

[12] Rabin R , de Charro F . EQ‐5D: a measure of health status from the EuroQol group. Ann Med. 2001;33(5):337‐343. 11491192 10.3109/07853890109002087

[13] Davies MG , Saad WE , Peden EK , Mohiuddin IT , Naoum JJ , Lumsden AB . Impact of runoff on superficial femoral artery endoluminal interventions for rest pain and tissue loss. J Vasc Surg. 2008;48(3):619‐625. discussion 625‐616. 18727964 10.1016/j.jvs.2008.04.013

[14] Bakken AM , Palchik E , Hart JP , Rhodes JM , Saad WE , Davies MG . Impact of diabetes mellitus on outcomes of superficial femoral artery endoluminal interventions. J Vasc Surg. 2007;46(5):946‐958. discussion 958. 17980281 10.1016/j.jvs.2007.06.047

[15] Shammas AN , Jeon‐Slaughter H , Tsai S , et al. Major limb outcomes following lower extremity endovascular revascularization in patients with and without diabetes mellitus. J Endovasc Ther. 2017;24(3):376‐382. 28440113 10.1177/1526602817705135 PMC5624231

[16] Jones WS , Patel MR , Tsai TT , et al. Anatomic runoff score predicts cardiovascular outcomes in patients with lower extremity peripheral artery disease undergoing revascularization. Am Heart J. 2015;170(2):400‐408. 26299239 10.1016/j.ahj.2015.04.026

[17] Chen J , Mohler ER 3rd , Garimella PS , et al. Ankle brachial index and subsequent cardiovascular disease risk in patients with chronic kidney disease. J Am Heart Assoc. 2016;5(6):e003339. 27247339 10.1161/JAHA.116.003339 PMC4937276

[18] Anand SS , Bosch J , Eikelboom JW , et al. Rivaroxaban with or without aspirin in patients with stable peripheral or carotid artery disease: an international, randomised, double‐blind, placebo‐controlled trial. Lancet. 2018;391(10117):219‐229. 29132880 10.1016/S0140-6736(17)32409-1

[19] Armstrong EJ , Chen DC , Westin GG , et al. Adherence to guideline‐recommended therapy is associated with decreased major adverse cardiovascular events and major adverse limb events among patients with peripheral arterial disease. J Am Heart Assoc. 2014;3(2):e000697. 24721799 10.1161/JAHA.113.000697 PMC4187469

[20] Hess CN , Wang TY , Weleski Fu J , et al. Long‐term outcomes and associations with major adverse limb events after peripheral artery revascularization. J Am Coll Cardiol. 2020;75(5):498‐508. 32029132 10.1016/j.jacc.2019.11.050

[21] Smith ME , Sutzko DC , Beck AW , Osborne NH . Provider trends in Atherectomy volume between office‐based laboratories and traditional facilities. Ann Vasc Surg. 2019;58:83‐90. 30684609 10.1016/j.avsg.2018.12.059

