# Supplementary material for: The impact of chronic kidney disease on outcomes following peripheral vascular intervention
Source: Clin Cardiol. 2020 Aug 11;43(11):1308–16. doi: 10.1002/clc.23444 (PMC7661640; doi:10.1002/clc.23444)
Supplement: Supplementary file 1 — Supplemental Table 1 Analysis of covariates used in adjusted model for MAVE [file CLC-43-1308-s001.docx]

Supplemental Table 1. Analysis of covariates used in adjusted model for MAVE

|  | Univariable | | Multivariable | |
| --- | --- | --- | --- | --- |
|  | HR (95% CI) | p Value | HR (95% CI) | p Value |
| Rutherford 2-3 vs. 6^†^ | 0.33 (0.23, 0.48) | <0.0001 |  |  |
| Prior lower limb endovascular treatment on target limb | 1.97 (1.58, 2.46) | <0.0001 | 1.93 (1.54, 2.42) | <0.0001 |
| Rutherford | NA^*^ | <0.0001 | NA^*^ | <.0001 |
| Rutherford 2-3 vs. 4-5^†^ | 0.57 (0.45, 0.73) | <0.0001 |  |  |
| History of previous lower limb peripheral endovascular treatments | 1.61 (1.28, 2.03) | <0.0001 |  |  |
| Previous amputation on target limb | 2.06 (1.38, 3.08) | 0.0004 |  |  |
| Rutherford 4-5 vs. 6^†^ | 0.58 (0.41, 0.81) | 0.0015 |  |  |
| Diabetes Mellitus | 1.47 (1.15, 1.86) | 0.0017 |  |  |
| History of Coronary artery disease | 1.43 (1.12, 1.81) | 0.0035 |  |  |
| History of Myocardial infarction | 1.36 (1.07, 1.73) | 0.0136 | 1.35 (1.06, 1.73) | 0.0160 |
| Smoker (Current/Former vs Never) | 0.77 (0.61, 0.97) | 0.0260 |  |  |
| ABI (compressible vs non-compressible) | 0.70 (0.50, 0.98) | 0.0356 |  |  |
| Prior lower limb bypass surgery on target limb | 1.61 (1.00, 2.59) | 0.0509 |  |  |
| History of Hypertension | 1.42 (0.85, 2.39) | 0.1811 |  |  |
| Race (Non-White vs White) | 1.16 (0.88, 1.54) | 0.2845 |  |  |
| Age (1 year increase) | 1.00 (0.99, 1.01) | 0.4612 |  |  |
| BMI (1 unit increase) | 0.99 (0.98, 1.01) | 0.5895 |  |  |
| History of Stroke/TIA | 1.05 (0.77, 1.43) | 0.7727 |  |  |
| Gender (Female vs. Male) | 0.97 (0.77, 1.23) | 0.8096 |  |  |
| History of Hyperlipidemia | 0.99 (0.71, 1.37) | 0.9354 |  |  |

This table displays a Cox proportional hazards model using stepwise selection with an entry criterion from the univariable model of 0.15 and a stay criterion of 0.05. The significant variables found here were used as covariates in an adjustment model for analyzing patients with CKD versus those without CKD for the composite outcome major adverse vascular events (MAVE).

^*^ Type III p-values displayed for categorical variables with more than 2 levels (hazard ratio=NA)

^†^Contrast statement used to estimate Hazard Ratio between 2 levels of Rutherford Category
